# Addressing and Overcoming Barriers to E-Cigarette Use for Smoking Cessation in Pregnancy: A Qualitative Study

**DOI:** 10.3390/ijerph17134823

**Published:** 2020-07-04

**Authors:** Katharine Bowker, Michael Ussher, Sue Cooper, Sophie Orton, Tim Coleman, Katarzyna Anna Campbell

**Affiliations:** 1 Division of Primary Care, School of Medicine, University of Nottingham, Nottingham NG7 2RD, UK; sue.cooper@nottingham.ac.uk (S.C.); sophie.orton@nottingham.ac.uk (S.O.); Tim.coleman@nottingham.ac.uk (T.C.); kasia.campbell@nottingham.ac.uk (K.A.C.); 2 Population Health Research Institute, St George’s, University of London, London SW17 0RE, UK; mussher@sgul.ac.uk; 3 Institute for Social Marketing and Health, University of Stirling, Stirling FK9 4LA, UK

**Keywords:** pregnancy, electronic cigarettes, vaping, smoking cessation, barriers

## Abstract

E-cigarettes may have a role in supporting pregnant women who would otherwise smoke to stop smoking. The study aimed to understand pregnant women’s vaping experiences, in particular how vaping to stop smoking is facilitated and how barriers to this are overcome. We conducted semi structured telephone interviews (*n* = 15) with pregnant or postpartum women who vaped during pregnancy, either exclusively (*n* = 10) or dual-used (*n* = 5) (smoked and vaped). Thematic analysis was used to analyse the interviews. Two themes emerged. First, ‘facilitating beliefs’: inherent beliefs that helped women overcome barriers to vaping. These included understanding the relative safety of vaping and economic gains compared with smoking and pregnancy being a motivator to stop smoking. Second, ‘becoming a confident vaper’: accumulating sufficient skill and confidence to comfortably vape. This included experimentation with e-cigarettes to ensure nicotine dependence and sensory needs were met. Seeking social support and employing strategies to address social stigma were also important. Positive beliefs about vaping and becoming proficient at vaping were viewed as ways to overcome barriers to vaping. The theoretical domain framework informed intervention recommendations to assist pregnant smokers who have tried but cannot stop smoking to switch to vaping.

## 1. Introduction

Smoking in pregnancy causes considerable harm, including increased risk of low birthweight, pre-term delivery and stillbirth [1,2]. Globally, it is estimated that 2% of women smoke during pregnancy, although this varies considerably between countries [3]. In England 10.5% of women report smoking at the time of their delivery [4] and smoking rates are much higher amongst younger and more deprived women [5]. Many women who smoke often try to stop smoking during pregnancy but are unsuccessful in their quit attempt, and those that manage to stop often return to smoking in the postpartum [6]. Finding effective ways to help pregnant women quit smoking and remain abstinent in the long-term is an important public health priority.

Electronic cigarettes (e-cigarettes/vaping) are often used by non-pregnant smokers to stop or cut down smoking [7]. Cross sectional studies from the US and UK show the prevalence rate of vaping in pregnancy to be between 0.6% and 15% [8,9,10,11]. Qualitative research suggests that some pregnant women believe e-cigarettes can be a helpful smoking cessation aid [12,13,14,15,16], yet many pregnant vapers also continue to smoke (dual users) [8,9]. Research identifies several barriers to vaping in pregnancy, including concerns about the safety of e-cigarettes and lack of reliable information about potential harm to the unborn baby [13,16], as well as perceived social stigma around vaping [13,17]. Cost and issues around using the device were also mentioned as problematic [13,16]. Women who experienced lack of support towards vaping were also reluctant to use them [13,18], while those who received positive messages were more likely to consider vaping [13,14,17,19].

Outside of pregnancy, a large randomised control trial has shown that in non-pregnant smokers, e-cigarettes were more effective than nicotine replacement therapy (NRT) for quitting smoking [20]. Biomarker studies, among non-pregnant individuals, show significantly lower levels of harmful chemicals among vapers who have stopped smoking than among smokers [21]. There are few studies which address the safety of vaping during pregnancy, but some indicate a potential association with adverse infant outcomes in women who dual use or vape excessively (50–70 times a day) [22,23]. A longitudinal study which compared the birthweight of the offspring of smokers, exclusive vapers and non-smokers found that the birthweight of babies born to women who vape and do not smoke was similar to non-smokers and higher than smokers [24].

E-cigarettes share a similar pharmacokinetic profile to NRT, where there are no harmful products of combustion [21,25,26]. However, like NRT, e-cigarettes often contain nicotine, and animal studies suggest that nicotine has a detrimental impact on pregnancy, and in human pregnancy, evidence on the effects of nicotine is very limited [27]. There is no evidence that nicotine from NRT has detrimental effects on the fetus or offspring [27,28,29], and although there are potential risks associated with nicotine use during pregnancy, in comparison to continued smoking it is deemed a safer alternative [30]. As many pregnant women find it difficult to quit smoking using traditional support such as NRT or behavioural support, e-cigarettes may have a role to help pregnant women stop smoking, who would otherwise continue to smoke. In the UK, although e-cigarettes are not risk free [31], guidance for health professionals (HP) is that they are safer and preferable to smoking, even for pregnant women [32].

Supporting pregnant women who vape and smoke (dual users) to completely switch to vaping, although not risk free, is likely to be a safer alternative to smoking [32]. We aim to describe how women who vape during pregnancy overcome potential or experienced barriers to vaping. Understanding the solutions women employ to overcome barriers and the facilitators of vaping may help health professionals to support pregnant dual users to stop smoking. This insight could also help develop interventions to support pregnant smokers switch from smoking to vaping in women who would otherwise continue to smoke.

## 2. Materials and Methods

Favourable ethical opinion was given in May 2019 by the Faculty of Medicine and Health Sciences Research Ethics Committee, University of Nottingham, reference 3191904. Two lay advisors, who previously smoked in pregnancy, reviewed the study materials, including the study advert and participant information sheets. We used the consolidated criteria for reporting qualitative research (COREQ) tool [33] to report the methods and findings.

### 2.1. Recruitment and Screening

Participants were purposively sampled to include pregnant women who self-report as exclusive vapers or dual users (smoke and vape). Recruitment occurred via adverts on Facebook and the Babycentre website (https://www.babycentre.co.uk/) [34], targeting specific demographics (i.e., 16–44 years old, female, interests in pregnancy/parenthood, living in the UK). Online adverts included study information and a link to an online screening questionnaire to determine eligibility. Eligible women were over 16, pregnant or ≤6 months postpartum and vaped during pregnancy for at least one week. Women were also asked whether they smoked while vaping, and those eligible were asked to provide their contact details.

Eligible women received a participant information sheet (PIS) via email explaining the purpose of the study. Recruitment was tailored to ensure a sample representative of both exclusive and dual vapers. Potential participants were contacted by telephone to arrange an interview and to provide an opportunity to ask questions. Informed consent was obtained verbally. Participants received a £15 shopping voucher as a thank you for their time.

### 2.2. Interviews

In-depth, semi-structured telephone interviews were used, and a qualitative descriptive methodology [35] was chosen to help understand how women overcome potential or experienced barriers to vaping in pregnancy. Author KB conducted telephone interviews between August and October 2019. Interviews were audio recorded and transcribed verbatim by an external transcriber.

Interview questions (Supplementary Materials S1 and S2) were based on research which identified barriers to vaping in pregnancy [13]. The key barriers to vaping which were discussed were social stigma, side effects, nicotine addiction, safety during pregnancy, difficulties with the device, cravings, cost and lack of information. The questions were primarily designed to elicit ideas on what solutions women employ to overcome these barriers, but also allowed participants to communicate new barriers to vaping. The interview questions were tailored to the two groups of e-cigarette users. The questions were also guided by the Theoretical Domains Framework (TDF), an integrative behaviour change framework which can aid understanding of key life domains wherein barriers and facilitators to change lie [36,37]. The TDF has been incorporated into previous work exploring smoking cessation in pregnancy [38].

After 15 interviews, we concluded that data saturation had been reached in both groups [34]. This was achieved by contemporaneously analysing the interviews while conducting the interviews, enabling the researcher to continually reflect and assess whether there were new themes to explore and develop. We interviewed more exclusive vapers, as this group appeared to have more experiences of employing solutions to barriers than dual users.

### 2.3. Analysis

The study is situated in the interpretive paradigm [39]. Thematic analysis was used to identify and report patterns and themes within the data [40]. Inductive analysis was used to explore women’s solutions to overcome barriers, but the analysis was guided by existing literature on barriers to vaping. KB coded all the transcripts and established the initial coding frame. Author KAC coded 33% of the transcripts using the coding frame. The two researchers discussed the coding framework and its coherence; the initial codes were reordered by importance, combined where appropriate, and grouped into themes. The researchers made notes where there were discrepancies within themes and took care to include all views. The TDF was used to align the codes and themes. During coding, we considered any potential differences between exclusive vapers and dual users and we identified no major differences. NVivo 11 software (QSR International Pty Ltd., released 2015, Melbourne, Australia) assisted with the coding.

All authors are researchers with backgrounds in midwifery, psychology, primary care and/or public health with significant experience of smoking cessation in pregnancy. None are smokers/vapers. The research group is within the UK, where health authorities support e-cigarette use in pregnancy for women unable to quit smoking by other means [32], which may have affected the authors’ views. Data collection and analysis was conducted by KB and KAC, both PhD qualified, females with experience in qualitative research. A lay advisor who had experience of smoking in pregnancy reviewed the themes and extracts from the data, to ensure that the messages being delivered in the paper were appropriate and meaningful to the original data.

## 3. Results

The Facebook advert was viewed 35,224 times by 8033 targeted Facebook users; 113 women selected the online survey. We are not able to report the number of women who viewed the Babycentre advert or who went on to click the link. A total of 72 women completed the online survey, of which 45 were eligible for an interview; 28 were exclusive vapers and 17 were dual users.

We interviewed 15 of the 45 eligible women, the remaining 30 women were either not contactable or not contacted as data saturation had been reached. Of the women interviewed, eight were pregnant and seven postpartum. Ten were exclusive vapers during pregnancy, and five were dual users. Participants were 22–36 years old, white British and the majority had a partner and were employed; 14 women were educated to GCSE level and above. Women were geographically spread over the UK: Scotland *n* = 1, Northern Ireland *n* = 1, Isle of Wight *n* = 1 and the north *n* = 3, south *n* = 4 east *n* = 2 and midland *n* = 4 regions of England. Interviews lasted for a mean of 31 m (range 19–43).

We identified two major themes. The first was ‘facilitating beliefs’, which were inherent beliefs that women held which helped them overcome barriers to vaping; this included the following sub themes: understanding the relative safety of vaping compared with tobacco smoking, believing vaping made economic sense and being motivated by pregnancy. The second theme was ‘gaining confidence as a vaper’, which was about accumulating a sufficient level of skill and confidence to comfortably vape. This included the following subthemes: experimentation, seeking or experiencing social support, actively seeking confirmation about e-cigarette safety and employing strategies to address social stigma. Table 1 shows the number of participants who contributed to each theme.

### 3.1. Theme 1: Facilitating Beliefs

#### 3.1.1. Subtheme 1.1: Understanding the Relative Safety Compared to Tobacco Smoking

Women felt that vaping was safer than smoking, even in pregnancy. They were aware that e-cigarettes contained nicotine but often concluded that compared with smoking they contained less dangerous ingredients. Holding strong beliefs about the relative safety of vaping enabled women to feel confident about their decision to vape, as they felt it was the safest option for them and their unborn baby.

“*I just feel that because there’s, obviously there’s nicotine but because there’s not all the other harmful chemicals in it, I feel it’s a lot better for yourself and the baby than smoking, so I guess nothing is better, but it’s better than still craving the cigarettes and wanting it, like, smoking them than it is to have something that’s got a lot less bad stuff in it.*”

Exclusive user (ID, 2)

Most vapers viewed e-cigarettes as a way to reduce and eventually end their addiction to nicotine. Unlike tobacco cigarettes, women felt by using e-cigarettes they could control the nicotine they consumed and limit exposure to nicotine over time. Dual users often reported vaping to cut down or wean off cigarettes gradually. Exclusive vapers reported vaping to stop smoking; they sometimes reported trying to wean off nicotine too, by lowering the nicotine level in their e-cigarette solution with the intention of quitting vaping. Some of the exclusive vapers had stopped using nicotine when vaping, and a few had subsequently ceased vaping. One exclusive vaper, who reported being addicted to vaping, felt strongly that more support was required to assist women to cease vaping.

#### 3.1.2. Subtheme 1.2: Economic Sense

Most women felt that vaping was cheaper compared to smoking and that the up-front costs of vaping paid off.

“*You can get sort of like a cheaper device that’s sort of like 20, 30 quid. But when you think about that, if you think about the cost of a packet of cigarettes and how long you’re going to be using it for, the cost of the vape and the juice and coils and everything, the cost compared to smoking is minimal.*”

Exclusive user (ID, 7)

Some women felt it could become costly if the device they chose wasn’t suitable or if they found it difficult to find a suitable nicotine strength or flavour, as they would need to experiment. Starter kits were suggested, to simplify the purchasing process, as they provided all the equipment a new vaper would require and were good value. Women varied in their view of how much to pay for devices. If money was tight, some women suggested trying supermarket versions, which could be cheaper than branded products. However, others believed you get what you pay for, and avoided cheap devices.

“*Perhaps look for your cheaper supermarket versions of the pens and not go for—if you—there’s, like, stores and stuff in, like, our shopping centre and you’re looking on about, sort of, £30 to £40 for very similar, but because it’s sort of the—whatever company they’re from, because it’s their brand and then you’re paying sort of about £6 for the liquids as well. And I think, you know, it is pretty much the same but because you’ve got someone there demonstrating it for you and it’s their brand, they charge you a lot more.*”

Exclusive user (ID, 2)

A few women believed that choosing to vape was a long-term investment, believing that e-cigarettes were a safer option for their own health and hopefully for their child’s health too. This was a strong motivation and without e-cigarettes they would struggle to stop smoking.

“*But it is cheaper than cigarettes and it’s obviously investing for your health long term isn’t it?*”

Exclusive user (ID, 13)

#### 3.1.3. Subtheme 1.3: Pregnancy as a Motivator

Pregnancy was a strong motivator for women to stop smoking; often women would think about the health effects of smoking on their pregnancy and feel guilty about the possible damage. Women vaped either to cut down or quit in pregnancy in order to protect their baby from the harmful effects of smoking. By holding on to the reasons why they were vaping, which was often for their baby, it helped women feel determined to cut down or quit smoking.

“*And I think if you’re strong enough to follow through and pursue with it and set your mind to it and think ‘hang on this is a healthier option for me and my baby’, then you can actually conquer the cravings.*”

Exclusive user (ID, 15)

“*I just knew that I had to, I just knew that…and I knew that even when I wasn’t pregnant, when I’ve got a young baby, smoking around them isn’t the right thing to do and even though, you know, vaping around them isn’t the best thing to do either, at least they’re not breathing in the chemicals, you know, they’re not passive smoking*”

Dual user (ID, 6)

### 3.2. Theme 2: Gaining Confidence as a Vaper

#### 3.2.1. Subtheme 2.1: Experimentation

Some women felt it was important to experiment with vaping devices, nicotine dose and flavours, in order to ensure it was satisfying nicotine cravings, providing an appealing sensory experience and limiting negative side effects. A few women reported that a sore throat or cough after vaping was the result of too high a dose of nicotine and after reducing the dose their side effects resolved. Women found that having a family member or friend who had vaped was helpful, as they could provide advice about using and maintaining the device and they could practice with their devices.

“*To be honest my husband, if anything goes wrong with it [e-cigarette) he tends to fiddle with it and fix it any way, he’s pretty good. He uses his far more than I use mine. So yeah, he knows what he’s doing with it, I just hand it over.*”

Exclusive vaper (ID, 13)

Women also recommended using vape shops, who they felt gave helpful practical advice. Acting as a consumer and researching the available products helped women take ownership of vaping and feel confident about taking action if difficulties occurred, for example changing flavour or device, rather than returning to smoking. One woman felt cautious towards vape shops, believing their motive was to make a profit. When starting vaping, some women described needing to learn to vape; the time it took for cravings to be alleviated and patterns of use were different as they often needed to vape more frequently compared with smoking.

“*It’s a completely experience to smoking, you do sort of have to learn how to use them I think because it’s not the same as smoking.*”

Dual user (ID, 6)

Some women stressed the importance of remembering to carry the e-cigarette around with them at all times and to keep the e-cigarette battery charged, to avoid any need for cigarettes. The patterns of use overtime changed for some exclusive vapers; they reported that the frequency they vaped often reduced, which they believed was a result of a reduced addiction to nicotine.

“*It has changed over time but to begin with I’d use it a lot, so I’d sit at home and I’d be like using it constantly, but I sort of don’t use it as much anymore.*”

Exclusive User (ID, 11)

#### 3.2.2. Subtheme 2.2: Seeking or Experiencing Social Support

Support from family, friends and work colleagues helped increase motivation and encouraged vaping among many women. A few women reported that family changed their smoking as a mark of support; for example, one woman reported her family no longer smoked when she visited.

Knowing friends and family who had vaped was important, as women felt vapers were more likely to understand their difficulties and could provide advice.

“*Everyone was really supportive. They said it was great that I was managing to quit because quite a lot of my family use e-cigarettes so they’ve had experience with it.*”

Exclusive user (ID, 7)

Online vaping forums provided support and women found other vapers’ experiences and advice helpful. Some women found health professionals (HP), usually midwives, supportive of their vaping and a few reported receiving praise and encouragement.

“*I was quite worried about using one but then also I felt it was better than cigarettes and my midwife actually praised me for it because she said you know, all my readings come up as the carbon monoxide and everything was all zeros, so she said to me ‘obviously that shows that it’s better to use one of them than it is to smoke cigarettes.*”

Exclusive user (ID, 2)

Most women felt that HP were supportive of stopping smoking but needed to be more supportive of vaping.

#### 3.2.3. Subtheme 2.3: Actively Seeking Confirmation about E-cigarette Safety

Most women felt that there was limited research about the safety of vaping during pregnancy. Some also felt that HPs offered little advice on the safety of vaping. Many felt they had to put extra effort into staying up to date by actively seeking information from HPs and online resources. By gaining knowledge, women felt able to make an informed decision about vaping.

Women found comparing safety information for cigarettes with e-cigarettes helpful as it led women to believe vaping was the healthier option. Women often sought reassurance from HPs, wanting confirmation that vaping was a healthier alternative to smoking.

“*If it’s a choice between cigarettes and a vape, I’d take a vape every day of the week, it’s got to be better compared to what’s in a cigarette and there’s not a massive amount of research done on vaping so far. I mean I know a doctor, not in a university and he makes it when I said to him ‘what do you think about this?’ he said ‘I’d rather you do that any day of the week than stick a cigarette in your mouth’ and this was before I was pregnant he was like ‘that’s got to be 10 times better.*”

Exclusive user (ID, 12)

One exclusive vaper stated that if her midwife had not endorsed vaping, she would reconsider using them.

#### 3.2.4. Subtheme 2.4: Employing Strategies to Deal with Social Stigma

Most women felt that there was social stigma around vaping during pregnancy, similar to smoking. Despite this, most women had not experienced direct criticism; a few felt that their friends and family would judge them about vaping. In response, some women found that knowing the latest research about the safety of vaping helped them feel confident, so if they were criticised, they could relay this to critics.

“*I’ve stayed away from vaping in front of the like complete strangers and then when people did say ‘oh well, this is bad for you, is it not bad for the baby?’ I would explain to them ‘well actually no, I’ve spoken to the midwife, this is 10 times better.*”

Exclusive user (ID, 12)

Some women were cautious about who they told about their vaping, in order to avoid judgement. One woman hid her vaping from her parents to avoid their disapproval. Other women sometimes vaped in private.

“*I will sometimes sneak off……depending on where I am, if I can sort of sneak off or have some in the car before or whatever, then I will. But yeah no, it is difficult if I’m going to be out especially, because my parents don’t know so if I’m out with my parents all day then that’s particularly difficult, so I might just leave [child’s name] with them and just say ‘oh, I’m just going to go and have a look over here.*”

Exclusive user (ID, 13)

### 3.3. Themes within the Theoretical Domain Framework (TDF)

We identified seven TDF domains which aligned with our themes. Table 2 highlights the domains which were associated with overcoming potential or experienced barriers and suggestions of intervention designs which could assist pregnant smokers to switch to vaping.

## 4. Discussion

This study has described the solutions women employ to overcome potential or experienced barriers to vaping during pregnancy. Women vaped to help them reduce or stop smoking with the intention of improving their own and their baby’s health and wellbeing; by focusing on these reasons, women felt motivated and determined. Facilitating beliefs such as believing that vaping was safer in comparison to smoking enabled women to feel confident about their decision to vape. An understanding that vaping made financial sense, in comparison to smoking, was also a facilitating belief. Knowledge and positive beliefs about the safety of vaping helped equip women to combat social stigma. Women felt it was important to become confident at vaping, and a period of adaptation when switching from smoking to vaping was often required. Peer support from vapers was perceived as valuable, along with endorsement from HPs. These solutions fell under seven domains of the TDF: skills, environmental context and resources, social influences, knowledge, behavioural regulation, beliefs about capabilities and beliefs about consequences.

There were some study limitations. The interviews were conducted over the telephone and do not capture contextual information, such as body language and facial cues. However, telephone interviews were considered an appropriate option given the geographical spread of participants and that in pregnancy it is a busy time for women. Some women were interviewed postpartum about their experiences in pregnancy, but by limiting recruitment to six months postpartum, we feel the potential for recall bias was reduced. Recruitment occurred online, relying on two resources, and could alienate women who do not access these resources or do not have internet. However, there are benefits of recruiting through such methods, as we were able to interview women from various areas of the UK.

This study was conducted in the UK only, therefore, we are uncertain of the extent to which findings are transferable to other contexts. The majority of pregnant smokers in the UK are white British and with low educational qualifications [41]; our sample was all white British participants. However, they were more highly educated than a representative population of smokers; this may be due to the online recruitment method [42] and that women who stop smoking during pregnancy tend to be more highly educated [41]. Future studies should consider targeting adverts to encourage a more representative sample. We also did not collect specific details about the type of e-cigarette product women used, or their vaping and smoking history, and therefore, we are not able to describe these. We purposively sampled exclusive and dual vapers. However, dual users were harder to recruit. We reflected on the interviews throughout data collection and found that not only were findings between the groups similar, but those who were exclusive users were often able to provide a greater insight into the solutions to barriers. Therefore, we decided to interview more women from the exclusive group.

To our knowledge this is the first qualitative study to describe the solutions pregnant women employ to overcome barriers to vaping in pregnancy. A previous qualitative study found that pregnant smokers sometimes viewed vaping as swapping one addiction for another [13]. However, we found that women believed that vaping could assist with reducing their nicotine addiction and smoking cessation. Women often reported that they reduced the nicotine content in their vape over time and some exclusive vapers had eventually managed to stop vaping too. It could be that e-cigarettes enable women to first substitute the sensory experience of smoking, and once this is mastered, women are able to combat their addiction to nicotine, making the experience of stopping smoking appear more manageable. Similar to non-pregnant vapers, we found that in order to maximise the vaping experience, a period of experimentation was required to substitute sensory differences by changing their vaping device and in some cases accepting vaping couldn’t completely substitute the smoking experience [43].

Similar to previous studies during pregnancy, women believed vaping was safer than smoking [13,16,17], and we found such beliefs were a facilitator to vaping. This is despite there being very limited data available about the fetal impacts of e-cigarette use during pregnancy. Seeking the latest information about vaping safety, through online sources or via HPs, and holding strong beliefs about the safety of vaping compared to smoking helped women to rationalise and feel confident about their decision to vape. Studies have found pregnant women feel concerned about the amount of nicotine delivered by products such as NRT [15,16] and e-cigarettes [13]. We found women were able to minimise these concerns by acquiring knowledge which enabled them to weigh up the risks of smoking compared with vaping. In addition, believing they had control over the nicotine they used in an e-cigarette helped minimize perceived risk.

Social support and positive recognition from friends, family or HPs provided a sense of validation that vaping was a healthier choice than smoking. Support from those who had vaped was particularly important, as women felt vapers were more likely to understand their challenges and offer important advice. The support of a HP was highly regarded by some vapers. However, women also reported that if a HP were not to endorse e-cigarettes, it would make them reconsider their decision to vape. Such findings are similar to views expressed by pregnant women around NRT [44,45].

Our findings have been incorporated into the TDF model, which can be used to inform e-cigarette interventions and support women who have not been able to quit smoking through traditional forms of support and would otherwise smoke. Our recommendations include interventions to disseminate knowledge about vaping, so that women can make informed decisions about vaping, especially compared with smoking. Interventions to assist women with the practicalities of vaping and managing expectations may also be helpful. HPs such as midwives have a unique relationship with women during pregnancy and, therefore, could play a fundamental role in interventions which support women who haven’t been able to quit through other means and would otherwise continue to smoke throughout pregnancy and postpartum. We also recognise the importance of support from other vapers; therefore, the use of peer support and involvement of family members could also be influential. Interventions to support women who vape exclusively should also be considered, so that women can stop addictions to both smoking and vaping.

## 5. Conclusions

The findings have highlighted how women overcome and manage barriers to vaping during pregnancy. Positive beliefs about vaping, particularly in comparison to smoking and becoming a confident vaper, were viewed as ways to overcome potential or experienced barriers to vaping. Pregnant smokers should first be supported to stop smoking through traditional forms of smoking cessation support. However, our research findings may help develop interventions to assist women who have tried to stop smoking but have not quit and would otherwise continue to smoke during pregnancy.

## Figures and Tables

**Table 1:** Summary of participants contribution to each theme.

Theme and Subtheme	Number of Participants Contributing to Theme. Total *n* = 15
**Facilitating beliefs**	
Understanding the relative safety of vaping compared with tobacco smoking	11/15
Believing vaping made economic sense	12/15
Pregnancy as a motivator	7/15
**Gaining confidence as a vaper**	
Experimentation	13/15
Seeking or experiencing social support	13/15
Actively seeking confirmation about e-cigarette safety	12/15
Employing strategies to address social stigma	11/15

**Table 2:** Theoretical Domain Framework and the identified themes.

Theme	Theoretical Domain Framework (TDF)	Recommendations for Interventions
*Understanding the relative safety compared to tobacco smoking*	***Beliefs about consequences*** *: believing that vaping was safer than smoking minimised women’s concerns about the harmfulness of vaping. Increased determination and confidence to vape.*	*Women require information about the safety and costs (economic and health) of vaping in comparison to smoking. Intervention delivery could include leaflets, websites, text messaging support and HP discussions.*
*Economic sense*	***Environmental context and resources*** *: women need to be able to afford the upfront costs associated with vaping; remembering they will be better off financially in the long run was important.* ***Beliefs about consequences*** *: immeasurable gains as vaping is beneficial for health and wellbeing in the long term.*	
*Actively seeking confirmation about the safety of e-cigarettes.*	***Knowledge*** *: the process of seeking and accumulating knowledge was important for women to validate their reasons for vaping.* ***Social influences*** *: HPs’ endorsement of the safety of vaping in comparison to smoking was important for continued use.*	
*Employing strategies to deal with social stigma*	***Social influences*** *: women felt social pressure not to vape during pregnancy.* ***Knowledge*** *: being able to defend their decision to vape was empowering.* ***Behavioural regulation*** *: to fit in with social norms, women often hid their vaping or took actions to only vape in situations they felt comfortable with.*	*Women require support to develop self-confidence/belief in their decision to vape as method to help them stop smoking. Interventions could include HP encouragement and showing the benefits of stopping smoking such as regular carbon monoxide readings.*
*Pregnancy as a motivator*	***Beliefs about capabilities*** *: determination and self-belief that they have the ability to change their smoking behaviour was crucial.* ***Beliefs about consequences*** *: women felt guilty about smoking; they worried about the effects on their baby and this was a motivator to vape rather than smoke.*	
*Experimentation*	***Skills*** *: through practice women acquired the skills to vape. This required time and persistence.* ***Environmental context and resources*** *: access to vape shops was a source of advice.* ***Social influences*** *: family and friends who were vapers were able to assist with choosing, experimenting and maintaining e-cigarettes.*	*Women need to develop practical skills to successfully vape. Intervention delivery could include peer support and HPs disseminating practical advice.*
*Seeking or experiencing social support*	***Social influences*** *: supportive friends, family and HPs increased motivation. Peer support from other vapers was regarded highly.*	*Women who cannot quit* via *other methods require support to vape in order to quit smoking. Interventions could involve peer support groups and involving family members in discussions with HPs about vaping.* *Support is also required for vapers who have stopped smoking but also want to stop vaping too.*

## Supplementary Materials

The following are available online at https://www.mdpi.com/1660-4601/17/13/4823/s1, Supplementary Materials S1: Interview schedule for exclusive vapers, Supplementary Materials S2: Interview schedule for dual users.

## References

[1] Delpisheh A., Kelly Y., Rizwan S., Attia E., Drammond S., Brabin B.J. (2007). Population attributable risk for adverse pregnancy outcomes related to smoking in adolescents and adults. Public Health.

[2] Cnattingius S. (2004). The epidemiology of smoking during pregnancy: Smoking prevalence, maternal characteristics, and pregnancy outcomes. Nicotine Tob. Res..

[3] Lange S., Probst C., Rehm J., Popova S. (2018). National, regional, and global prevalence of smoking during pregnancy in the general population: A systematic review and meta-analysis. Lancet Glob. Health.

[4] NHS Digital (2020). Statistics on Women’s Smoking Status at Time of Delivery: England Quarter 3, 2019–2020. https://digital.nhs.uk/data-and-information/publications/statistical/statistics-on-women-s-smoking-status-at-time-of-delivery-england/statistics-on-womens-smoking-status-at-time-of-delivery-england-quarter-3-2019-20.

[5] McAndrew F., Thompson J., Fellows L., Large A., Speed M., Renfrew M.J. (2012). Infant Feeding Survey 2010: Health and Social Care Information Centre. http://digital.nhs.uk/catalogue/PUB08694.

[6] Cooper S., Orton S., Leonardi-Bee J., Brotherton E., Vanderbloemen L., Bowker K. (2017). Smoking and quit attempts during pregnancy and postpartum: A longitudinal UK cohort. BMJ Open.

[7] West R., Beard E., Brown J. (2019). Trends in electronic cigarette use in England. http://www.smokinginengland.info/sts-documents/.

[8] Kapaya M., D’Angelo D.V., Tong V.T., England L., Ruffo N., Cox S., Warner L., Bombard J., Guthrie T., Lampkins A. (2019). Use of Electronic Vapor Products Before, During, and After Pregnancy Among Women with a Recent Live Birth—Oklahoma and Texas, 2015. MMWR Morb. Mortal. Wkly. Rep..

[9] Liu B., Xu G., Rong S., Santillan D.A., Santillan M.K., Snetselaar L.G., Bao W. (2019). National Estimates of e-Cigarette Use Among Pregnant and Nonpregnant Women of Reproductive Age in the United States, 2014–2017. JAMA Pediatr..

[10] Kurti A.N., Redner R., Lopez A.A., Keith D.R., Villanti A.C., Stanton C.A., Gaalema D.E., Bunn J.Y., Cepeda-Benito A., Roberts M.E. (2017). Tobacco and nicotine delivery product use in a national sample of pregnant women. Prevent. Med..

[11] Mark K.S., Farquhar B., Chisolm M.S., Coleman-Cowger V.H., Terplan M. (2015). Knowledge, Attitudes, and Practice of Electronic Cigarette Use Among Pregnant Women. J. Addict. Med..

[12] Ashwin C., Watts K. (2010). Exploring the views of women on using nicotine replacement therapy in pregnancy. Midwifery.

[13] Bowker K., Orton S., Cooper S., Naughton F., Whitemore R., Lewis S., Bauld L., Sinclair L., Coleman T., Dickinson A. (2018). Views on and experiences of electronic cigarettes: A qualitative study of women who are pregnant or have recently given birth. BMC Pregn. Child..

[14] Bauld L., Graham H., Sinclair L., Flemming K., Naughton F., Ford A. (2017). Barriers to and facilitators of smoking cessation in pregnancy and following childbirth: Literature review and qualitative study. Health Technol. Assess..

[15] Bowker K., Campbell K.A., Coleman T., Lewis S., Naughton F., Cooper S. (2016). Understanding Pregnant Smokers’ Adherence to Nicotine Replacement Therapy During a Quit Attempt: A Qualitative Study. Nicotine Tob. Res..

[16] England L.J., Tong V.T., Koblitz A., Kish-Doto J., Lynch M.M., Southwell B.G. (2016). Perceptions of emerging tobacco products and nicotine replacement therapy among pregnant women and women planning a pregnancy. Prevent. Med. Rep..

[17] Grant A., Morgan M., Gallagher D., Mannay D. (2018). Smoking during pregnancy, stigma and secrets: Visual methods exploration in the UK. Women Birth.

[18] Bauld L., Graham H., Sinclair L., Flemming K., Naughton F., Ford A., McKell J., McCaughan D., Hopewell S., Angus K. (2017). Chapter 11. Perceptions of new interventions from three prespectives: Pregnant and postpartum women, their significant others and health-care professionals. Barriers to and Facilitators of Smoking Cessation in Pregnancy and Following Childbirth: Literature Review and Qualitative Study.

[19] Fallin A., Miller A., Assef S., Ashford K. (2016). Perceptions of Electronic Cigarettes Among Medicaid-Eligible Pregnant and Postpartum Women. J. Obst. Gynecol. Neonat. Nurs..

[20] Hajek P., Phillips-Waller A., Przulj D., Pesola F., Smith K.M., Bisal N., Li J., Parrott S., Dawkins L., Ross L. (2019). E-cigarettes compared with nicotine replacement therapy within the UK Stop Smoking Services: The TEC RCT. Health Technol. Assess..

[21] Shahab L., Goniewicz M.L., Blount B.C., Brown J., McNeill A., Alwis K.U., Feng J., Wang L., West R. (2017). Nicotine, Carcinogen, and Toxin Exposure in Long-Term E-Cigarette and Nicotine Replacement Therapy Users: A Cross-sectional Study. Ann. Intern. Med..

[22] Cardenas V.M., Cen R., Clemens M.M., Moody H.L., Ekanem U.S., Policherla A., Fischbach L.A., Eswaran H., Magann E.F., Delongchamp R.R. (2019). Use of Electronic Nicotine Delivery Systems (ENDS) by pregnant women I: Risk of small-for-gestational-age birth. Tob Induc Dis..

[23] Gillen S., Saltzman D. (2014). Antenatal exposure to e-cigarette vapor as a possible etiology to total colonic necrotizing enterocolitits: A case report. J. Pediatr Surg Case Rep..

[24] McDonnell B., Dicker P., Regan C. (2020). Electronic cigarettes and obstetric outcomes: A prospective observational study. BJOG Int. J. Obstetr. Gynaecol..

[25] Benowitz N.L., Burbank A.D. (2016). Cardiovascular toxicity of nicotine: Implications for electronic cigarette use. Trends Cardiovasc. Med..

[26] Dejmek J., Solansky I., Benes I., Lenicek J., Sram R.J. (2000). The impact of polycyclic aromatic hydrocarbons and fine particles on pregnancy outcome. Environ. Health Perspect..

[27] Wickström R. (2007). Effects of Nicotine During Pregnancy: Human and Experimental Evidence. Cur. Neuropharmacol..

[28] Ogburn P.L., Hurt R.D., Croghan I.T., Schroeder D.R., Ramin K.D., Offord K.P., Moyer T.P. (1999). Nicotine patch use in pregnant smokers: Nicotine and cotinine levels and fetal effects. Am. J. Obstet Gynecol..

[29] Wright L.N., Thorp J.M., Kuller J.A., Shrewsbury R.P., Ananth C., Hartmann K. (1997). Transdermal nicotine replacement in pregnancy: Maternal pharmacokinetics and fetal effects. Am. J. Obstet. Gynecol..

[30] Bar-Zeev Y., Lim L.L., Bonevski B., Gruppetta M., Gould G.S. (2018). Nicotine replacement therapy for smoking cessation during pregnancy. Med. J. Aust..

[31] American Lung Association (2020). What’s in an E-cigarette. https://www.lung.org/quit-smoking/e-cigarettes-vaping/whats-in-an-e-cigarette.

[32] Smoking in Pregnancy Challenge Group (2019). Use of Electronic Cigarettes Before, During and After Pregnancy. A Guide for Maternity and Other Healthcare Professionals. http://smokefreeaction.org.uk/wp-content/uploads/2019/07/2019-Challenge-Group-ecig-briefing-A4-FINAL.pdf.

[33] Tong A., Sainsbury P., Craig J. (2007). Consolidated criteria for reporting qualitative research (COREQ): A 32-item checklist for interviews and focus groups. Int. J. Qual. Health Care.

[34] BabyCentre (2019). Pregnancy, baby and toddler health information at BabyCentre UK. https://www.babycentre.co.uk/.

[35] Bradshaw C., Atkinson S., Doody O. (2017). Employing a Qualitative Description Approach in Health Care Research. Glob. Qual. Nurs. Res..

[36] Cane J., Richardson M., Johnston M., Ladha R., Michie S. (2015). From lists of behaviour change techniques (BCTs) to structured hierarchies: Comparison of two methods of developing a hierarchy of BCTs. Br. J. Health Psychol..

[37] Michie S., Atkins L., West R. (2014). The Behaviour Change Wheel: A Guide to Designing Interventions.

[38] Campbell K., Fergie L., Coleman-Haynes T., Cooper S., Lorencatto F., Ussher M. (2018). Improving Behavioral Support for Smoking Cessation in Pregnancy: What Are the Barriers to Stopping and Which Behavior Change Techniques Can Influence Them? Application of Theoretical Domains Framework. Int. J. Environ. Res. Public Health.

[39] Scotland J. (2012). Exploring the philosophical underpinnings of research: Relating ontology and epistemology to the methodology and methods of the scientific, interpretive, and critical research paradigms. Engl. Lang. Teach..

[40] Braun V., Clarke V. (2006). Using thematic analysis in psychology. Qual. Res. Psychol..

[41] Orton S., Bowker K., Cooper S., Naughton F., Ussher M., Pickett K.E., Leonardi-Bee J., Sutton S., Dhalwani N.N., Coleman T. (2014). Longitudinal cohort survey of women’s smoking behaviour and attitudes in pregnancy: Study methods and baseline data. BMJ Open.

[42] Emery L.J., Coleman T., Sutton S., Cooper S., Leonardi-Bee J., Jones M. (2018). Uptake of Tailored Text Message Smoking Cessation Support in Pregnancy When Advertised on the Internet (MiQuit): Observational Study. J. Med. Inter. Res..

[43] Hoek J., Thrul J., Ling P. (2017). Qualitative analysis of young adult ENDS users’ expectations and experiences. BMJ Open.

[44] Borland T., Babayan A., Irfan S., Schwartz R. (2013). Exploring the adequacy of smoking cessation support for pregnant and postpartum women. BMC Public Health.

[45] Mantzari E., Vogt F., Marteau T.M. (2012). The effectiveness of financial incentives for smoking cessation during pregnancy: Is it from being paid or from the extra aid?. BMC Preg. Child..

