# Supplementary material for: Addressing and Overcoming Barriers to E-Cigarette Use for Smoking Cessation in Pregnancy: A Qualitative Study
Source: Int J Environ Res Public Health. 2020 Jul 4;17(13):4823. doi: 10.3390/ijerph17134823 (PMC7369696; doi:10.3390/ijerph17134823)
Supplement: Supplementary file 1 [file ijerph-17-04823-s001.zip › S2- dual user interview.docx]

**Dual users interview schedule**

**Background questions about woman’s EC use during pregnancy**

- When did you first start using an EC?
  - - In pregnancy/or before?
    - Tell me how you came to use one?
    - *For those who have used an EC prior to pregnancy-* did becoming pregnant change the way you felt or used an EC?
- In what way did it change the way you felt/used an EC
- What type of device did you use?
  - - Why did you choose this one?
    - What strength of nicotine did you use, Why?
    - Did you use a flavour?
- How often did/do you use an EC in pregnancy?
  - - How often are you using it/or did you use it in pregnancy I.e. every day, many times a day? Describe your typical day?

**Barriers to stopping smoking using an EC**

- You have said that you smoke OR smoked, and you have/had *Cut down/smoked the same amount (based on online screening questions)* during pregnancy
  - - Can you tell me more about this?
      1. *Cut down*
         1. Was this your intention? If yes why? If no what changed, did you plan to quit?
         2. How did you find the EC helped you cut down?
         3. Why do you think you have not been able to stop smoking?
         4. Is there anything you feel could help you stop smoking?
      2. *Smoke the same*
         1. Was this your intention? If yes why? If no what changed, did you plan to cut down or quit?
         2. How have you found ECs help you?
         3. Do you want to stop smoking? If yes, is there anything you feel could help you stop smoking? If no, can you tell me why not?
- Did you discuss using an EC with anyone during pregnancy, in particular did you discuss dual use (so smoking and using an EC)?
  - - *If Yes*- can you tell me more about this? Who from? When?
    - *If no*- why not? What support and advice would you have liked?
- How do you decide between whether to have an EC or smoke during the day?
  - - What prevents you from using an EC rather than smoking?
    - What makes you choose to use your EC over smoking?
    - Can you think of anything that does or could make you choose to use an EC over a cigarette?

*We want to find out about any difficulties or problems you experienced when using your EC in pregnancy, and most importantly we want to know how you overcame the issues you experienced or what you think would have helped you.*

- Pregnant women sometimes worry about how others may react to them using an EC during pregnancy. For example they would feel uncomfortable using an EC in public while pregnant.
  - What are your thoughts about this?
  - Was this an issue for you?
  - If yes,
    - Do you think this influenced how you used your EC?
    - How did you deal with this? Can you describe how you overcame this?
    - Did you receive any support
      - If yes can you tell me more, was this enough? What more could have helped?
      - If no, what support would you have wanted? How do you think this would have helped?
    - What advice would you give to other pregnant women about overcoming this issue?
- Pregnant women sometimes report they have experienced side effects after using ECs
  - What are your thoughts about this?
  - Was this an issue for you?
  - If yes,
    - Do you think this influenced how you used your EC?
    - How did you deal with this? Can you describe how you overcame this?
    - Did you receive any support
      - If yes can you tell me more, was this enough? What more could have helped?
      - If no, what support would you have wanted? How do you think this would have helped?
    - What advice would you give to other pregnant women about overcoming this issue?
- Pregnant women sometimes worry that using an EC does not get rid of nicotine addiction
  - What are your thoughts about this?
  - Was this an issue for you?
  - If yes,
    - Do you think this influenced how you used your EC?
    - How did you deal with this? Can you describe how you overcame this?
    - Did you receive any support
      - If yes can you tell me more, was this enough? What more could have helped?
      - If no, what support would you have wanted? How do you think this would have helped?
    - What advice would you give to other pregnant women about overcoming this issue?
- Pregnant women sometimes report that there is not enough information on the safety of ECs, specifically their safety in pregnancy
  - What are your thoughts about this?
  - Was this an issue for you?
  - If yes,
    - Do you think this influenced how you used your EC?
    - How did you deal with this? Can you describe how you overcame this?
    - Did you receive any support
      - If yes can you tell me more, was this enough? What more could have helped?
      - If no, what support would you have wanted? How do you think this would have helped?
    - What advice would you give to other pregnant women about overcoming this issue?
- Pregnant women sometimes report that ECs might be as harmful as cigarettes
  - What are your thoughts about this?
  - Was this an issue for you?
  - If yes,
    - Do you think this influenced how you used your EC?
    - How did you deal with this? Can you describe how you overcame this?
    - Did you receive any support
      - If yes can you tell me more, was this enough? What more could have helped?
      - If no, what support would you have wanted? How do you think this would have helped?
    - What advice would you give to other pregnant women about overcoming this issue?
- Pregnant women sometimes report that they have experienced difficulties using their device or don’t get on with their device
  - What are your thoughts about this?
  - Was this an issue for you?
  - If yes,
    - Do you think this influenced how you used your EC?
    - How did you deal with this? Can you describe how you overcame this?
    - Did you receive any support
      - If yes can you tell me more, was this enough? What more could have helped?
      - If no, what support would you have wanted? How do you think this would have helped?
    - What advice would you give to other pregnant women about overcoming this issue?
- Pregnant women sometimes report that they don’t feel that their EC is helping with their cravings for a cigarette
  - What are your thoughts about this?
  - Was this an issue for you?
  - If yes,
    - Do you think this influenced how you used your EC?
    - How did you deal with this? Can you describe how you overcame this?
    - Did you receive any support
      - If yes can you tell me more, was this enough? What more could have helped?
      - If no, what support would you have wanted? How do you think this would have helped?
    - What advice would you give to other pregnant women about overcoming this issue?
- Pregnant women sometimes report they think it’s too costly to use an EC
  - What are your thoughts about this?
  - Was this an issue for you?
  - If yes,
    - Do you think this influenced how you used your EC?
    - How did you deal with this? Can you describe how you overcame this?
    - Did you receive any support
      - If yes can you tell me more, was this enough? What more could have helped?
      - If no, what support would you have wanted? How do you think this would have helped?
    - What advice would you give to other pregnant women about overcoming this issue?
- Pregnant women have found that there is not enough information on how to use ECs, for example advice on which device or solution to use?
  - What are your thoughts about this?
  - Was this an issue for you?
  - If yes,
    - Do you think this influenced how you used your EC?
    - How did you deal with this? Can you describe how you overcame this?
    - Did you receive any support
      - If yes can you tell me more, was this enough? What more could have helped?
      - If no, what support would you have wanted? How do you think this would have helped?
    - What advice would you give to other pregnant women about overcoming this issue?
- Pregnant women sometimes feel they would like more support and advice from health professionals
  - What are your thoughts about this?
  - Was this an issue for you?
  - If yes,
    - Do you think this influenced how you used your EC?
    - How did you deal with this? Can you describe how you overcame this?
    - Did you receive any support
      - If yes can you tell me more, was this enough? What more could have helped?
      - If no, what support would you have wanted? How do you think this would have helped?
    - What advice would you give to other pregnant women about overcoming this issue
- Some pregnant women have reported they have experienced negative opinions and comments from friends and family about using an EC during pregnancy
  - What are your thoughts about this?
  - Was this an issue for you?
  - If yes,
    - Do you think this influenced how you used your EC?
    - How did you deal with this? Can you describe how you overcame this?
    - Did you receive any support
      - If yes can you tell me more, was this enough? What more could have helped?
      - If no, what support would you have wanted? How do you think this would have helped?
    - What advice would you give to other pregnant women about overcoming this issue
- Can you describe any other difficulties you have experienced while using your EC that you think has stopped you or stopped you wanting to use your EC, or meant that you decided to smoke instead of vape?
  - Please tell me more about this
  - Did you overcome this issue?
    - If yes, how?
    - If no, why not? What could have helped you? How do you think it would have helped?
- Can you think of anything else that could help pregnant women to quit smoking while using ECs?
  - - What do you think could help them?
    - What support is required? Who is best to give this?

*Thank you for you answers to these questions, is there anything further you wish to add?*

*Finally I just want to ask you a few quick questions about yourself…*

**Ask about:**

- How many weeks pregnant are you or How many weeks postpartum
- Age
- Ethnicity
- What is the highest qualification you hold?
- Are you currently employed?
- Do you have a partner? Married?
  1. Do they smoke?
